# Supplementary material for: Coexistence with Pseudomonas aeruginosa alters Staphylococcus aureus transcriptome, antibiotic resistance and internalization into epithelial cells
Source: Sci Rep. 2019 Nov 12;9:16564. doi: 10.1038/s41598-019-52975-z (PMC6851120; doi:10.1038/s41598-019-52975-z)
Supplement: Supplementary file 1 — Supplementary data [file 41598_2019_52975_MOESM1_ESM.pdf]

Coexistence with *Pseudomonas aeruginosa* alters *Staphylococcus aureus* transcriptome, antibiotic resistance and internalization into epithelial cells

Paul Briaud, Laura Camus, Sylvère Bastien, Anne Doléans-Jordheim, François Vandenesch and Karen Moreau.

Supplementary data

Supplementary data

Table S1: Laboratory *S. aureus* strains, other species strains and plasmids used in this study.

Strains and Plasmids		Genotypes	References
S. aureus	LUG3040	RN6390 WT	[2]
	LUG2955	RN6390 Δ*tet38*	This study
	LUG26	Newman WT	[3]
	LUG1790	Newman *RNAIII*::*tetM*	[4]
	LUG30	Newman *agr*::*tetM*	[5]
	LUG3135	Newman *mgrA*::*tetM*	[6]
	LUG2262	Lac WT	[7]
	LUG3134	Lac *ΔmgrA*	[6]
	LUG2995	SF8300 WT	[8]
	LUG2987	SF8300 *ΔagrA*	[8]
B. cepacia	LUG2886	CF clinical strains	This study
S. maltophilia	LUG2884	CF clinical strains	This study
Plasmids	pMAD	pMAD vector	[1]
	pMAD-*tet38*	pMAD for *tet38* allelic exchange	This study

1. Arnaud, M., Chastanet, A. & Debarbouille, M. New Vector for Efficient Allelic Replacement in Naturally Nontransformable, Low-GC-Content, Gram-Positive Bacteria. *Appl. Environ. Microbiol.* **70**, 6887–6891 (2004).
2. Novick, R. P. *et al.* Synthesis of staphylococcal virulence factors is controlled by a regulatory RNA molecule. *EMBO J.* **12**, 3967 (1993).
3. Duthie, E. S. & Lorenz, L. L. Staphylococcal coagulase; mode of action and antigenicity. *J. Gen. Microbiol.* **6**, 95–107 (1952).
4. Supersac, G., Piémont, Y., Kubina, M., Prévost, G. & Foster, T. J. Assessment of the role of gamma-toxin in experimental endophthalmitis using ahlg-deficient mutant ofStaphylococcus aureus. *Microb. Pathog.* **24**, 241–251 (1998).
5. Wolz, C., McDevitt, D., Foster, T. J. & Cheung, A. L. Influence of agr on fibrinogen binding in Staphylococcus aureus Newman. *Infect. Immun.* **64**, 3142–3147 (1996).
6. Crosby, H. A. *et al.* The Staphylococcus aureus Global Regulator MgrA Modulates Clumping and Virulence by Controlling Surface Protein Expression. *PLoS Pathog.* **12**, (2016).
7. Voyich, J. M. *et al.* Is Panton-Valentine Leukocidin the Major Virulence Determinant in Community-Associated Methicillin-Resistant Staphylococcus aureus Disease? *J. Infect. Dis.* **194**, 1761–1770 (2006).
8. Rasigade, J.-P. *et al.* PSMs of Hypervirulent Staphylococcus aureus Act as Intracellular Toxins That Kill Infected Osteoblasts. *PLoS ONE* **8**, (2013).

Table S2: List of primers used in this study

Primers	Direction	Sequences (5'-3')	Target	Size (bp)
OLUG4-F	Forward	AGCGAGTGAAAACACGCAAC	*sbi*	152
OLUG4-R	Reverse	TTCTTGTGCACGTTCTGGGT		
OPB1bF	Forward	CAAACTGCCGGTTTAGCTGC	*norb_3*	103
OPB1bR	Reverse	TGCTCGTACTTAAGCCAAGGT		
OPB2F	Forward	ACAATTTGGCACACCAACAGA	*potA*	100
OPB2R	Reverse	TCTAACCATGCGTCCTTCAACA		
OPB5F	Forward	ACGCAAGAAGAACTTGCTGAAC	*potR*	124
OPB5R	Reverse	GCGTCGTTCCTAACACCTCT		
OPB19bF	Reverse	CAACACGTTGCCATTGTTGC	*nrdE*	149
OPB19bR	Forward	GACACTAGCTCACCACGACG		
OPB24F	Reverse	TCCGCCAGCTAAGTTCCAAG	*sirA*	142
OPB24R	Forward	TCCAAATGCGAAAGATGCTGC		
OPB31F	Reverse	AACGTTGGATGCGTATGGGT	*deoD*	88
OPB31R	Forward	AATGCTTCGACACCAGCGTA		
OPB32F	Reverse	AGCTGAAGCGACTTTGTCAGATGC	*mgrA*	110
OPB32R	Forward	AGCGTGAACGTTCCGAAGTCGA		
SarZ-F1	Forward	GATTCTGGAACACTGACACCAT	*sarZ*	138
SarZ-R1	Reverse	AGCAAGAGGGCTTTTATTGCT		
OPB35F	Forward	CAATTGGAACGCACGAGTCAA	*tetR21*	176
OPB35R	Reverse	GGGCTGTTTGTCCATTACCCA		
OLUG1-F	Forward	GGTGGCGACTTTGATCTAGC	*gyrB*	169
OLUG1-R	Reverse	TTATACAACGGTGGCTGTGC		
OLUG2-F	Forward	TTTACGTGCAGCACGTTCAC	*hU*	125
OLUG2-R	Reverse	AAAAAGAAGCTGGTTCAGCAGTAG		
Pr1-F	Forward	TATGACGTCCACGCGTGGTTGTGTT TGCGTCACG	*tet38 upstream*	981
Pr1-R	Reverse	CCCGGGAGCCGAATTCAAT CATCTACACCAATGACAGTGC		
Pr2-F	Forward	CTCCCGGGTACCATGGATAGAT ATAAATTCGCGAGAT	*tet38 dowstream*	1000
Pr2-R	Reverse	TTAACTAGACAGATCTCTCAAT TTAGTATAGTATGCTTCTG		
OPB40F	Forward	TATCGCCGTTTGGTGGTACG	*norA*	178
OPB40R	Reverse	GTCACACCCGGCATTACCAT		
OPB41F	Forward	TGGTCAATTGGCTCATGGGG	*norB*	78
OPB41R	Reverse	ACGCCAACCTAAAAGTGTTGC		
OPB42bF	Forward	AGTTGGCGTTGCTTCAGGTA	*norC*	87
OPB42bR	Reverse	CCGGCATATACTGCACCACT		
OPB21bF	Forward	CTGGAACGATGGAATGGGCT	*sstA*	114

OPB21bR	Reverse	ACGTACCGCAAATACTGCAA		
OPB48F	Forward	GGGCATTAGATGCGACAGCA	*sak*	70
OPB48R	Reverse	ACTTCGATCTTTGCGCTTGG		
OP45F	Forward	ATGGCCAGAGTTACGAGTGT	*nrdF*	93
OP45R	Reverse	CGTCATTTTCCCTTGACCAGC		
OPB49F	Forward	CCACTGCACTCGCACGATTA	*scn_3*	70
OPB49R	Reverse	TTGCTAGTTTTATCATTGGGAGCA		
HlaF	Forward	GGTAATGTTACTGGTGATGATACAGGAA	*hla*	72
HlaR	Reverse	TGCAAATGTTTCGATTGGTCATACAC		
saeR-F1	Forward	TGACCCACTTACTGATCGTGG	*saeR*	154
saeR-R1	Reverse	ACCGCTAGTTGTCGTTGTTACT		
OBP46F	Forward	GGGCTAAGTCTATTAGGTGGCG	*nrdD*	78
OPB46R	Reverse	ACGTGCTCGAAATGCTTTGAC		
OPB52F	Forward	TCACCAGCAGCATTAGCGAT	*aur*	93
OPB52R	Reverse	TGCTTTGACCGCATCACTCT		

Table S3: MIC of the 12 couples of strains

	MIC (μg/ml)	
Strains	Tetracycline	Ciprofloxacin
SA27	0,7	8
SA30	11,25	1
SA31	0,5	8
SA42	1	1
SA69	0,5	4
SA80	8	2
SA82	0,5	2
SA146	0,5	4
SA152	1	16
SA153	1	8
SA156	4	2
SA2599	4	4
PA27	8	2
PA30	8	1,5
PA31	12	0,5
PA42	4	0,047
PA69	1,5	1,5
PA80	4	0,38
PA82	8	8
PA146	24	0,38
PA152	16	1
PA153	12	2
PA156	8	0,19
PA2600	16	6

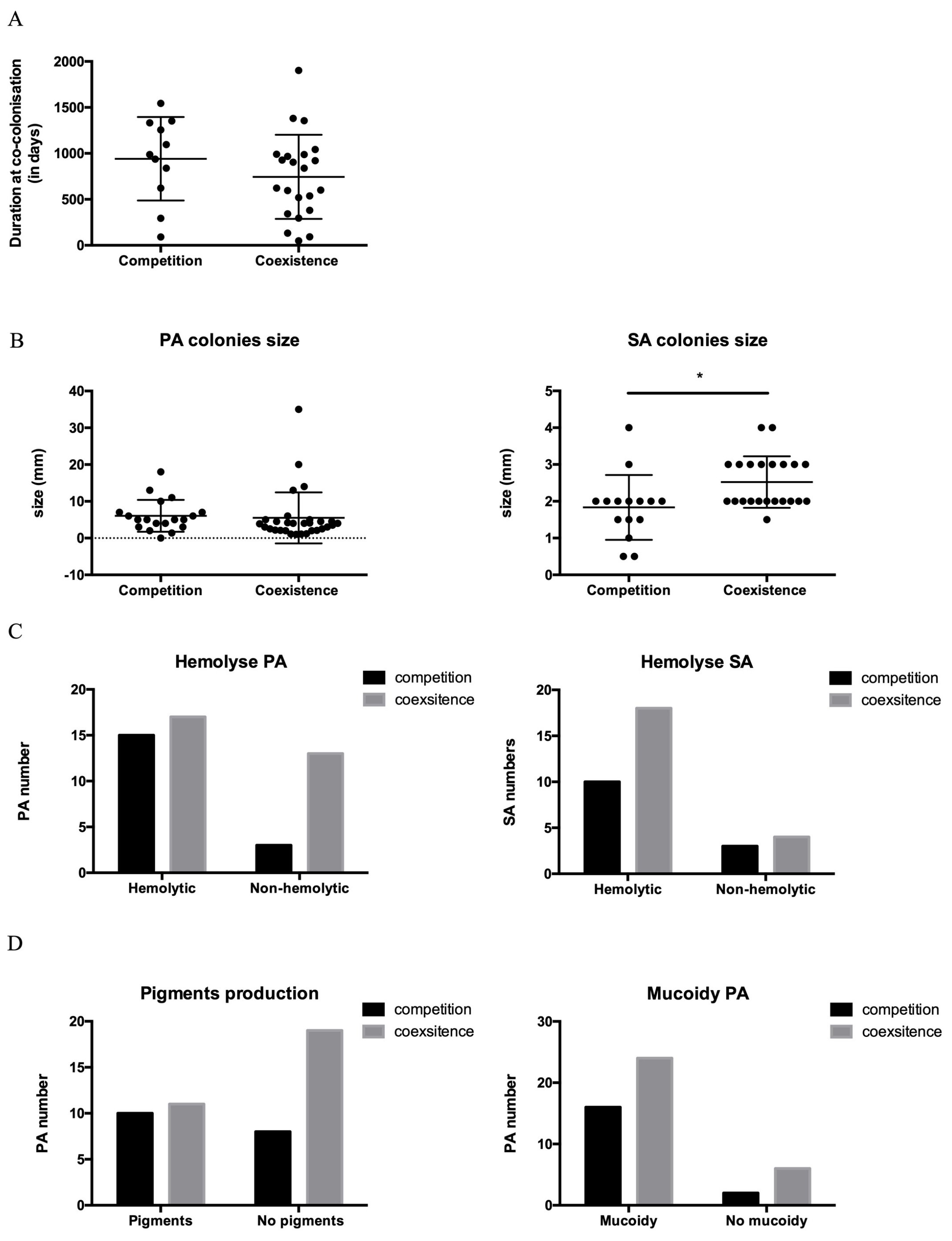

Figure S1: Statistical analysis of phenotypic differences between coexisting and competitive strains. No significant differences were observed for the duration of co-colonization (**A** – T-test, p value =0,2532), *P. aeruginosa* colonies size (**B**- T test, p-value for PA =0,7489 and for SA=0,0119), hemolytic properties (**C** – Fisher test, p-value for PA =0,0,0683 and for SA>0,999), *P. aeruginosa*

pigment production and mucoid phenotype (**D**- Fisher test, p-value for pigment =0,2402 and for mucoidy =0,6918). A significant difference was observed for *S. aureus* colony sizes.

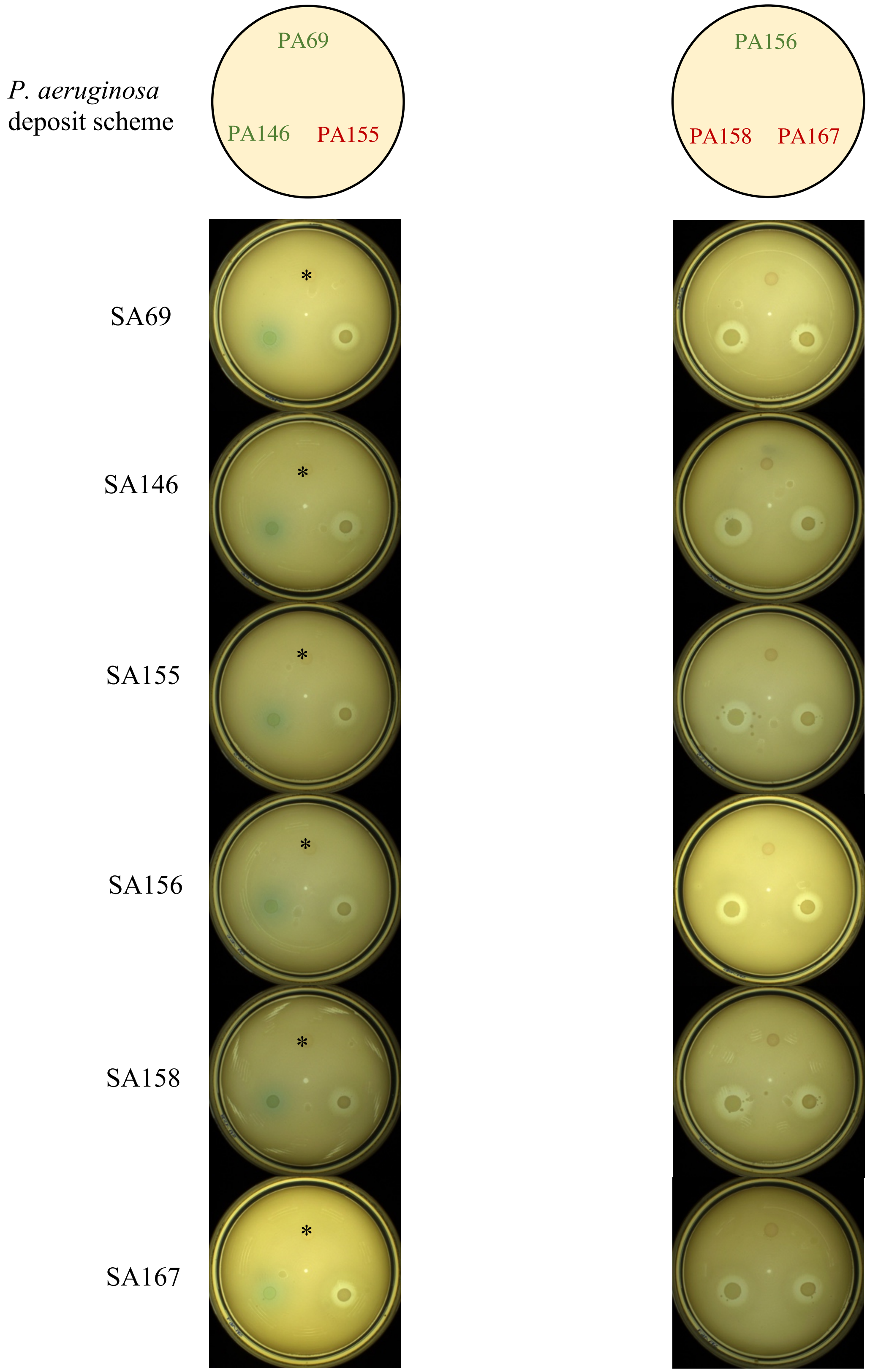

Figure S2: Crossed agar competition assay. *S. aureus* - *P. aeruginosa* strain pairs 69, 146, 155, 156, 158 and 167 were cultivated over-night in BHI at 37°C. *S. aureus* strains were plated at OD_{600nm}=0.5 onto TSA as indicated on the left of the photograph and left to dry for 15min. *P. aeruginosa* spots were deposited as depicted in the top scheme. *P. aeruginosa* green tags represent a coexisting isolate (absence of inhibition halo) whereas red tags represent a competition isolate (presence of inhibition halo). * symbols indicate the PA69 spots, which are faintly visible on the pictures.

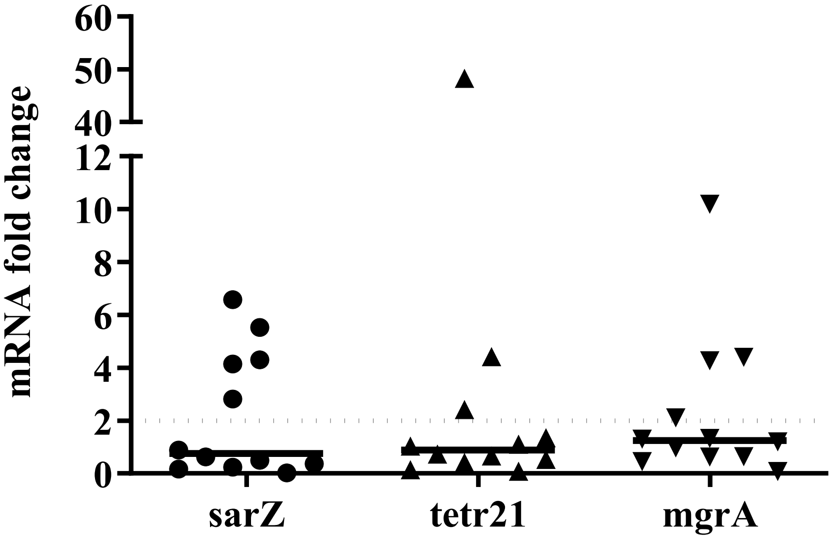

Figure S3: Coculture with *P. aeruginosa* has no effect on *tet38* major regulators. RNAs were extracted from a mono- and coculture at 4 hours and gene expression was monitored by RT-qPCR**.** The results represent the median gene expression obtained from twelve coexisting strain pairs. The dotted line depicts fold change= 2.

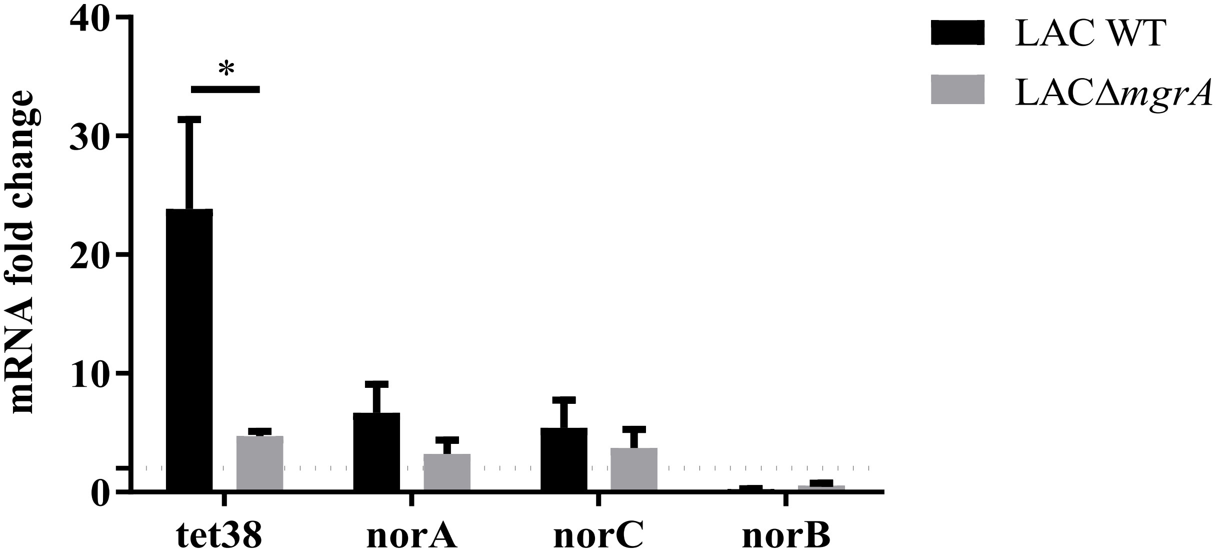

Figure S4: MgrA is important for *nor* genes over-expression. Cocultures with *S. aureus* LAC wild type (WT) and *mgrA* mutants (Δ*mgrA*) and PA30 were performed. RNAs were extracted at 8 hours and *nor* gene expression monitored by RT-qPCR. The dotted line indicates a fold change= 2. The results are shown as the mean + standard deviation of three independent experiments. Statistical analysis was performed by unpaired t-test (* P< 0.05)

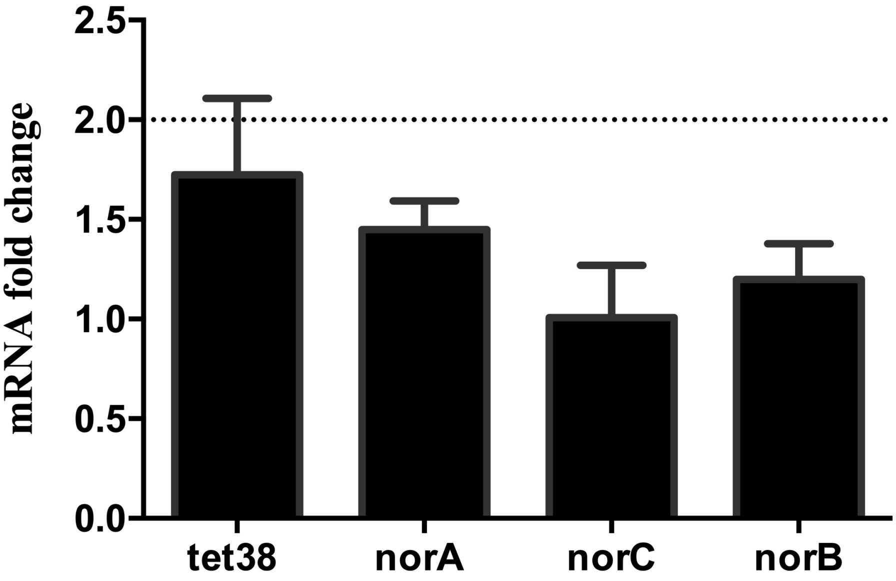

Figure S5: *S. aureus nor* genes over-expression requires close contact with *P. aeruginosa*. *S. aureus* was exposed to an 8 hours supernatant culture of *P. aeruginosa*. RNAs were extracted and *nor* gene expression was monitored by RT-qPCR. Dotted lines represent fold change= 2. The results are shown as the mean + standard deviation of three independent experiments on SA30-PA30 pairs. Statistical analysis was performed by unpaired t-test (* P<0.05, ** P<0.01).

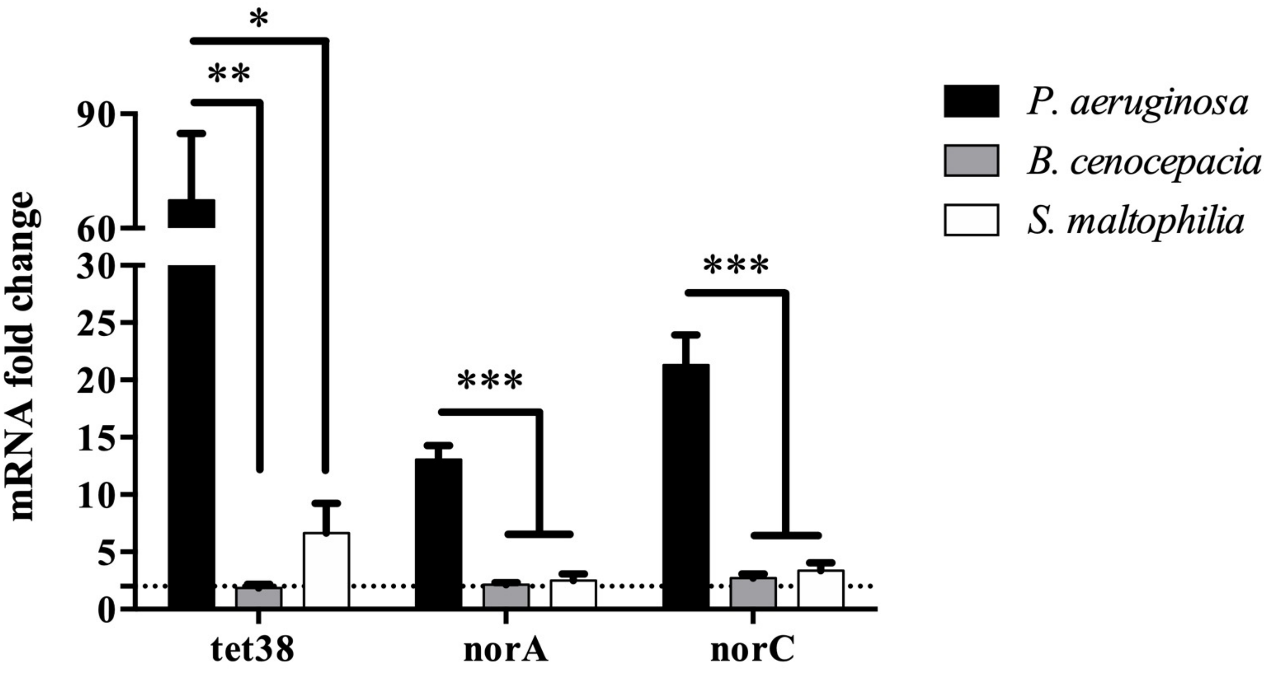

Figure S6: Specificity of *nor* genes family over-expression induced by *P. aeruginosa*. *S. aureus* was mono- and co-cultivated as described earlier. RNAs were extracted at 8 hours and gene expression was monitored by RT-qPCR. The dotted line indicates a fold change= 2. The results represent the mean + standard deviation of three independent experiments conducted with pair SA146-PA146. Statistical analysis was performed by One-way Anova with the Dunnett correction multiple test (* P< 0,05, ** P < 0,01, *** P<0,001).

A

	Pair 27	Pair 31	Pair 69
Mono-infection (SA)	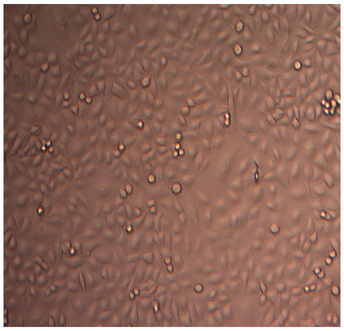	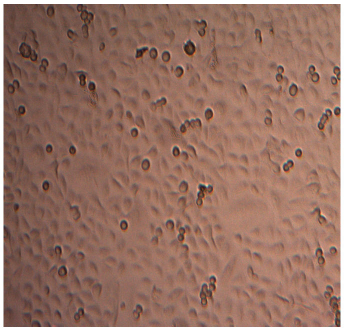	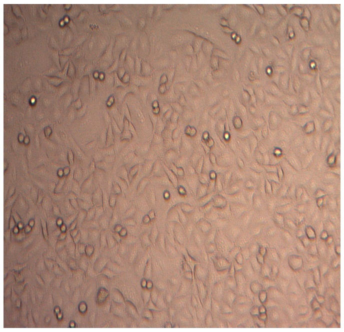
Co-infection (SA+PA)	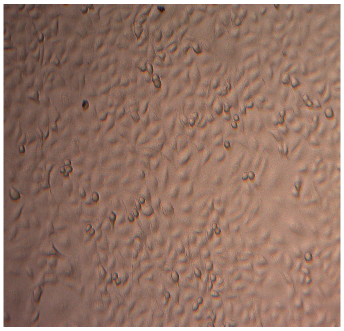	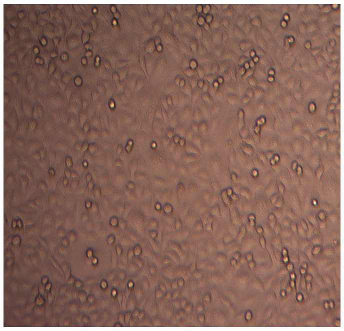	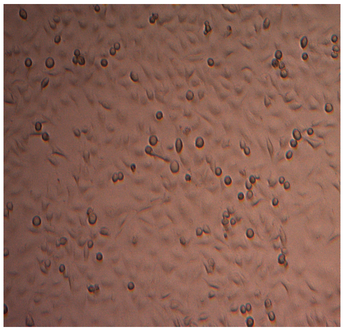
Non-Infected		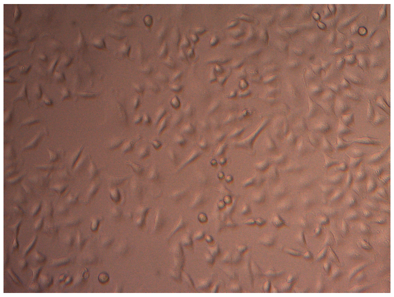	

B

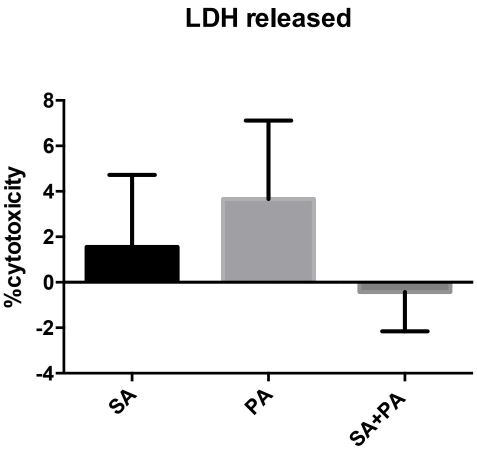

Figure S7: Co-infection with *S. aureus* and *P. aeruginosa* do not impact cell shape and viability. A549 cells were infected at a multiplicity of infection (MOI) of 10:1 for mono-culture and 20:1 for coculture. After 2 hours of infection and 1 hour of antibiotic treatment to eliminate extracellular bacteria, cells were photographed (**A**) and LDH was measured in the supernatant using CytoTox-ONE™ Homogeneous Membrane Integrity Assay (Promega) (**B**) following the manufacturer's instructions.
